# Phenolics Profile and Protective Effect on Injuried HUVEC Cells of Epicarp Extracts from *Kadsura coccinea*

**DOI:** 10.3390/foods11040556

**Published:** 2022-02-16

**Authors:** Jun Lu, Ying Zheng, Zhenyu Yang, Jing Cheng, Feijun Luo

**Affiliations:** 1 Hunan Key Laboratory of Forestry Edible Sources Safety and Processing, Central South University of Forestry and Technology, Changsha 410004, China; 20171200327@csuft.edu.cn (Y.Z.); 20201100407@csuft.edu.cn (Z.Y.); luofeijun888@csuft.edu.cn (F.L.); 2 National Engineering Research Center of Rice and By-Product Deep Processing, Central South University of Forestry and Technology, Changsha 410004, China; 3 Hunan Key Laboratory of Food Safety Science and Technology, Technology Center of Changsha Customs, Changsha 410004, China; chengjing@csc.intra.custom.gov.cn

**Keywords:** *Kadsura coccinea*, antioxidant activity, antioxidative, HUVEC

## Abstract

This study evaluated the phenolics profile and the antioxidative properties of *K. coccinea* fruits epicarp. A total of 13 phenolic compounds (six phenolic acids, four anthocyanins, two flavonols, and one flavone) were identified by ultra performance liquid chromatography coupled with quadrupole time-of-flight tandem mass spetrometry(UPLC-QTOF-MS/MS). Two anthocyanins, cyanidin-3-xylosylrutinoside and cyanidin-3-rutinoside, comprise 30.89~74.76% and 13.90~46.17% of the total amount of anthocyanins in *K. Coccinea*. Cytoprotective effect results evidenced that pretreatment of Human umbilical vein endothelial cells(HUVECs) with *Kadsura. coccinea* fruits’ epicarp phenolic extracts at the concentrations of 50–200 µg/mL improved the cell viability after exposure to H_2_O_2_ significantly, and inhibited malonaldehyde(MDA) and reactive oxygen species(ROS) overproduction, as well as enhancing the content of superoxide dismutase (SOD) and glutathione Reductase (GR. This study proved that *K. coccinea* is a natural resource of phenolics rich with potential antioxidant ability, which may be valuable for developing nutraceuticals and dietary supplements.

## 1. Introduction

Oxidative stress occurs due to the imbalance between reactive oxygen species (ROS) and antioxidants, resulting in the excessive production of various pro-inflammatory cytokines and promotion of inflammatory responses, subsequently to damage vital biological molecules cellular biofilm, DNA, protein and other substances, eventually leads to the occurrence of a variety of diseases, such as diabetes, cardiovascular disease, respiratory disease, rheumatoid arthritis and various cancers [1,2]. In recent years, natural products derived from fruits have gained a lot of attention as potential active ingredients against oxidative stress or chemoprevention agents, especially phenolic compounds, many studies suggest that the antioxidant capacity of natural plants is closely related to their total phenolic content and composition [3,4,5]. Numerous studies from nature resource have shown that phenolic compounds can reduce the level of oxidative stress and increase the antioxidant capacity in the body to prevent and reduce the occurrence of diseases related to oxidative stress [6,7,8]. So far, more than 8000 natural phenolic compounds have been identified. Therefore, many efforts have been dedicated to finding the role that natural oxidants play in alleviating oxidation, thereby preventing or delaying oxidative stress, and to find new sources of such compounds.

*Kadsura Coccinea* (Lem.) A.C. Smith commonly known as “heilaohu”, is an important medical plant belong to family Schisandraceae, with a long history in the western and southwestern China as a Chinese folk herbal medicine. It is also known as the “Magical Medicine of the Dong Nationality” because the Dong nationality ethnic group use the roots and stems to treat several illnesses such as gastric ulcer, rheumatism, sore throats and to relieve pain, and in dispelling flatulence [9] because the abundance of lignans and triterpenoids [10]. Nowadays, much of the research in *K. coccinea* has focused on the study of the chemical composition and the bioactive compounds of its root, thus resulting in diverse diterpenoids, triterpenoids and lignans gbeing identified. The *K. coccinea* fruit is a succulent edible fruit with a special shape and unique taste. To date, less research has been completed on its phenolic composition and corresponding antioxidant activity, especial for the epicarp, the larger edible parts in the fruit. Therefore, the aim of the present study is to determine their phenolics composition and evaluate the protective effect activity against cell oxidative damage. This experiment firstly evaluated some new phenolic compounds from *K. coccinea* and their protective effect on HUVEC oxidative stress induced by H_2_O_2._ The results showed that *K. coccinea* phenolic extracts administered pretreatment enhanced the antioxidant capacity and alleviated the cell injury caused by oxygen free radicals.

## 2. Materials and Methods

### 2.1. Materials and Chemicals

**Figure 1:**
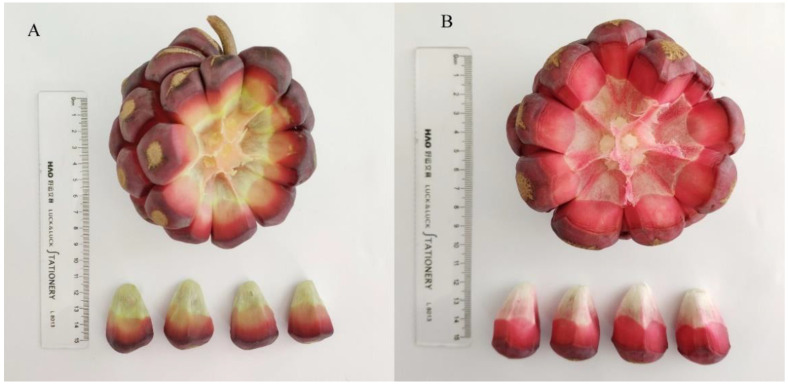
Two kinds of *K. Coccinea,* Zihe (**A**) and Dahong (**B**).

Two kinds of *K. coccinea* fruit (Zihe and Dahong in Figure 1) were collected from Tongdao County, Hunan Province, (26°54′ N, 109°42′ E), China. The scientific identification was performed by Zhonghai Li (College of Food Science and Engineer, Central South University of Forestry and Technology, Changsha, China). HUVECs were purchased from the Institute of Cell Resource Center, Chinese Academy of Science (Shanghai, China), the cell line originated from ATCC (American Type Culture Collection, Manassas, VA, USA). Cyanidin-3-glucoside, protocatechuic acid, *p*-hydroxybenzoic acid, cinnamic acid, syringic acid andvitexin, were purchased from Sichuan Victory Biological Technology Co., Ltd. (Chengdu, China). Ferulic acid, *p*-coumaric acid, vanillic acid, hyperoside and rutin were purchased from Shanghai Yuanye Bio-Technology Co., Ltd. (Shanghai, China). Fetal bovine serum was purchased from Bioind (Kibbutz Beit Haemek, 25115, Israel). Dulbecco’s modified Eagle’s medium (DMEM), penicillin, streptomycin and trypsin were purchased from Gibco BRL (Grand Island, NY, USA). Cytotoxicity and the proliferation assay kit was purchased from PROMEGA (Madison, WI, USA). All other chemicals were analytical grade and purchased from the China National Pharmaceutical Corporation (Shanghai, China).

### 2.2. Extraction and Isolation of Phenolic Compounds

The epicarp was manually stripped from the *K. coccinea* fruit and ground with a grinder. A certain quality of epicarp pulping was added with 70% of ethanol solution (containing 1% hydrochloric acid) according to the ratio of 1:20 g/mL. After ultrasonic extraction for 60 min in an ultrasonic cleaning machine (Jining, China) at 50 °C, the extracted solution was centrifuged at 4000 r/min for 8 min. The supernatants were condensed and removed to solvent under reduced pressure. After that, the concentrated solutions were purified with LS-46D macroporous and desorbed with methanol containing 0.1% HCl. Finally, the desorption solutions were concentrated to remove solvent under reduced pressure at 55 °C. The residue was defined as ZHP for Zihe phenolics extracts and DHP for Dahong phenolic extracts.

### 2.3. Determination of Total Phenolics, Total Flavonoids and Total Anthocyanin

The total phenolics content (TPC) were estimated by Folin–Ciocalteu assay based on the method of Bursal et al. [11]. Gallic acid was used as the reference standard and total phenolic content was expressed as milligrams gallic acid equivalents per gram of fresh weight sample (mg GAE/g). Total flavonoids content (TFC) was determined according to the aluminum chloride colorimetry method [12]. Catechin was used as the reference standard and the total flavonoids content was expressed as milligrams catechin equivalents per gram of fresh weight sample (mg CE/g). The anthocyanin content (TAC) in the extracts was measured using the pH differential method described by Lee et al. [13]. TAC was expressed as mg cyanindin-3-glucoside equivalents per gram of fresh weight sample (mg Gy-3-G/g). The anthocyanin content was calculated according to the following equation:

Total anthocyanins (mg Gy-3-G/mL) = A × MW × DF × 1000/(ε × 1)

where A is absorbance difference = (A_510_ − A_700_)_pH1.0_ − (A_510_ − A_700_)_pH4.5_; MW is molecular weight of cyanindin-3-glucoside = 449.20 g/mol; DF is the dilution factor; ε is the molar absorptivity of cyanindin-3-glucoside = 26,900 L/(mol cm); L, pathlength in cm; 1000 is factor for conversion from g to mg.

### 2.4. Determination of Antioxidant Activity

In this study, 1,1-diphenyl-2-picrylhydrazyl (DPPH), ABTS (2,2′-azino-bis (3-ethylbenzothiazoline-6-sulphonic acid) diammonium salt) and the Ferric reducing antioxidant power (FRAP) assay were used to evaluate the antioxidant activity of phenolic extracts. The DPPH radical scavenging activity was assayed according to described by Gorjanović et al. [14], with appropriate modifications. The ABTS assay was tested according to the method described by Oh et al. [15]. The FRAP assay was performed according to the method described by Lu et al. [16]. These antioxidant activities were all expressed as micromole trolox equivalents per gram of fresh weight sample (μmol TE/g).

### 2.5. UPLC-QTOF-MS/MS Analysis

A 1290 Infinity UPLC system (Agilent Technologies, Palo Alto, CA, USA) coupled to an Agilent G6495 Triple Quadrupole mass spectrometer (Agilent Technologies, Palo Alto, CA, USA) was used to identify the phenolic compounds. These compounds were separated on an Agilent Poreshell C18 (150 mm × 2.1 mm, 2.7 μm, Agilent, Waters, Milford, MA, USA) column operated at 35 °C. The eluent system employed was a combination of A (acetonitrile) and B (0.1% formic acid in water) at a flow rate of 0.3 mL/min. The gradient varied linearly from 5% to 95% B (*v*/*v*) over 15 min. The mobile phase gradient profile was as follows: 0–1.5 min, 97% B; 1.5–2.5, 80% B; 2.5–28 min, 40% B; 28–31 min, 40% B. The sample injection volume was 10 μL. Mass spectra were obtained in the positive and negative modes in a mass range between 100 and1500 Da with the following operating parameters: Air curtain air CUR: 30 PSI; Atomizing gas GAS1: 55PSI; Auxiliary gas GAS2: 60 PSI; Source temperature: 550 °C; Cumulative time: 0.2 s; De-cluster voltage: 70 V. Mass spectra acquisition and data analysis were processed with Masshunter Workstation B 04.00 software (Agilent Technologies). The identification was achieved by comparison of available standards and references.

### 2.6. Cell Culture and Cell Viability Assay

HUVECs were incubated in RPMI Medium 1640 containing 10% FBS and 1% double antibody (100 mg L^−1^ penicillin and 100 mg L^−1^ streptomycin) at 37 °C in a humidified atmosphere of 5% CO_2._ Cells in logarithmic growth phase were placed in 96-well plate at 4 × 10^3^ cells/mL, 100 μL. The H_2_O_2_ group cells were treated with H_2_O_2_ (20, 30, 40, 50, 60 μmol L^−1^), the ZHP and DHP group cells were treated *K. coccinea* epicarp extracts at the concentration of 25, 50, 100, 200 and 300 μg/mL, Control group cells were cultured with complete 100% medium. After incubation for 24 h, 10 μL of [3-(4,5-dimethylthiazol-2-yl)-5-(3-carboxymethoxyphenyl)-2- (4-sulfophenyl)-2H-tetrazolium (MTS) were added and continued to be cultured for 2 h. The absorption values of each well were measured using an automated microplate reader (SpectraMax i3X, Molecular Devices, Silicon Valley, CA, USA) at 490 nm. The data were calculated according to the following formula: percentage of cell viability = (OD of H_2_O_2,_ ZHP, DHP group/OD of control group) × 100.

### 2.7. Determination of Protective Effect on HUVEC Injuried by H_2_O_2_

HUVEC cells in logarithmic growth phase were adjusted to 4 × 10^3^ cells/mL and inoculated into 96-well plates. After 4 h incubation for the cells to grow to confluence, the different concentrations of *K. coccinea* extracts (50, 100 and 200 mg/mL) were added to the cells followed by another 10 h incubation. Then the cells were treated with H_2_O_2_ for 24 h after the original medium was removed. After that, the cell viability was measured by MTS assay.

### 2.8. Determination of ROS, MDA, SOD and GR

HUVEC cells in logarithmic growth phase were incubated in 6-well plates at a density of 1 × 10^5^ cells/mL. After 24 h of incubation, the cells were grouped as described in Section 2.6 and pretreatment *K. coccinea* extracts (50, 100 and 200 μg/mL) was performed for 24 h, followed by H_2_O_2_-induced injury for 24 h. Then, the level of ROS, MDA, SOD and GR in the cells was tested by commercially available kits according to the manufacturer’s instructions (Beyotime Biotechnology Co., Ltd., Shanghai, China). The results of SOD activity were expressed as a percentage (%) in the blank control group.

### 2.9. Statistical Analysis

Statistical analysis was analyzed through the software SPSS (version 20 Chicago, IL, USA). One-way analysis of variance (ANOVA) was performed and the significant differences on the results were determined by Tukey’s test at 5% significance level.

## 3. Results

### 3.1. The TPC, TFC, TAC and Antioxidant Capacity of K. coccinea Extracts

The results of TPC, TFC and TAC of two kinds of *K. coccinea* are shown in Table 1, the TPC in ZHP (2.56 ± 0.33 mg GAE/g) was lower than that of DHP (3.51 ± 0.14 mg GAE/g). However, the TFC and TAC in ZHP were significantly higher than that of DHP with the value of 5.94 ± 0.30 mg CE/g and 0.71 ± 0.02 mg Gy-3-G/g, respectively, especially for total anthocyanins in ZHP almost 3 times greater than DHP. The results of their chemical antioxidant activity also showed that there were significantly differences between the two kinds of *K. coccinea* extracts, except the DPPH radical scavenging activities of DHP exhibited higher activity than ZHP with the value of 111.57 ± 3.10 μmol TE/g, the ABTS and FRAP value all showed significantly lower levels than that of ZHP. The relative higher level of these three bioactive compounds in ZHP may be due to its dark black epicarp. As some previous reports indicated, the dark black color peel or epicarp usually exhibit abundant anthocyanins, especially the dark varieties, being responsible for the red, or violet to black colors [17,18].

### 3.2. The Effective Concentration of H_2_O_2_ and K. coccinea Extracts on HUVEC 

**Figure 2:**
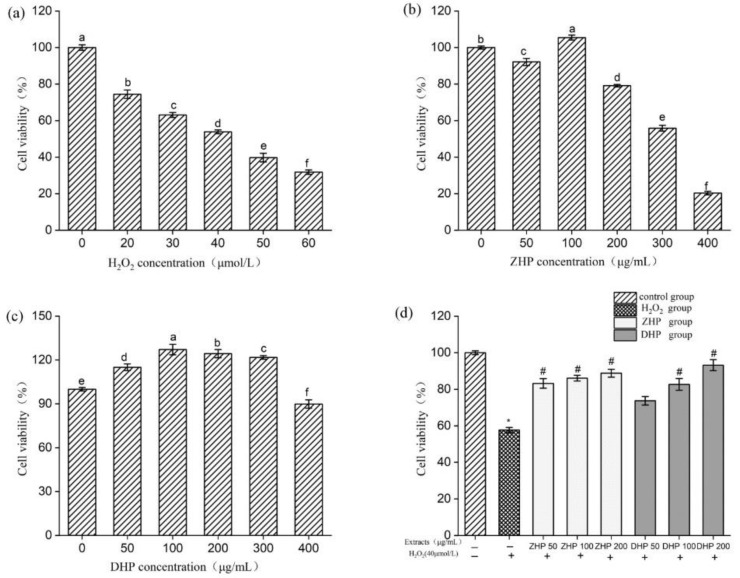
Effects of H_2_O_2_ (**a**); ZHP (**b**); and DHP (**c**) on cell viability of HUVEC. The protective effects of DHP and ZHP on cell viability after H_2_O_2_ injury (**d**). Values are presented as means ± SD (*n* ≥ 6); *p* < 0.05; * *p* < 0.05 vs. Control group. # *p* < 0.05 vs. H_2_O_2_ group.

Figure 2a shows the effective concentration of H_2_O_2_ and polyphenolics on HUVEC. The survival rate of HUVEC cells gradually decreased with the increase of H_2_O_2_ concentration (20–60 μmol/L), and decreased by 53.86% when exposed to 40 μmol L^−1^ of H_2_O_2_, when the H_2_O_2_ continued to increase to 50 and 60 μmol L^−1^, the cell survival rate decreased by 39.74% and 31.80%, respectively. In order to achieve the best conditions for the establishment of the cell oxidative damage model, the 54% was considered an appropriate survival rate for the continued test of the degree of cell damage, as the oxidative injury was recoverable under the impact of 40 μmol/L H_2_O_2_ solutions. Thus, concentration of 40 μmol L^−1^ of H_2_O_2_ was selected for the subsequent experiments. The non-toxic concentrations of *K. coccinea* extracts on cell viability were also determined using MTS and the results are shown in Figure 2b,c. At the treatment of 300 and 400 μg/mL of ZHP, the cell viability was decreased by 55.86% and 20.36%, respectively, but non-toxic effect was observed in DHP. Therefore, considering the cell death caused by the toxicity of the sample itself during the experiment, three concentrations of low (50 μg/mL), medium (100 μg/mL) and high (200 μg/mL) were selected for subsequent experiments. 

### 3.3. Effect of K. coccinea Extracts on Survival Rate of HUVEC after H_2_O_2_-Induced Injury

The protective effect of DHP and ZHP on cell viability of injured HUVEC is shown in Figure 2d. Under the treatment of 100 μmol/mL H_2_O_2_ without *K. coccinea* extracts, the cell survival decreased to 57.68%. On the contrary, after pretreatment with the *K. coccinea* extracts, the viability rate of HUVEC was markedly increased with a dose-dependent relationship. The viability rate of HUVEC cells increased by 35.50% and 31.13%, respectively, compared to the injured group when pretreatment with 200 μg/mL of ZHP and DHP, indicating that the extracts had a positive protective effect on HUVEC cells injured by H_2_O_2_ cytotoxicity.

### 3.4. Effect of K. coccinea Extracts on ROS of HUVEC after H_2_O_2_-Induced Injury 

**Figure 3:**
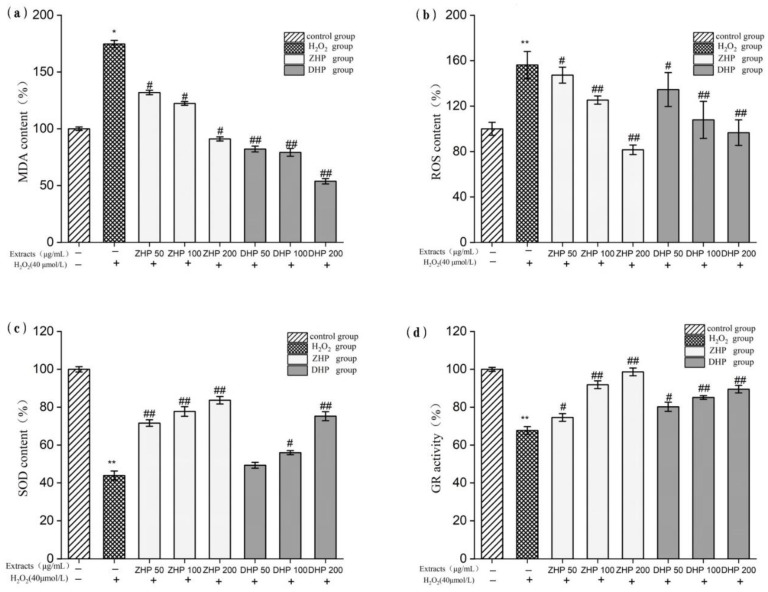
Effect of the DHP and ZHP on ROS content (**a**); MDA content (**b**); SOD activity (**c**); and GR activity (**d**) in HUVEC. Values are presented as means ± SD (*n* ≥ 6); * *p* < 0.05; ** *p* < 0.01 vs. Control group. # *p* < 0.05; ## *p* < 0.01 vs. H_2_O_2_ group.

The effect of *K. coccinea* extracts on ROS content in H_2_O_2_-injuried HUVEC cells is shown in Figure 3b, cells that were exposed to H_2_O_2_ stress had a significant increase in intracellular ROS (nearly 3-fold vs. the control group). However, stronger inhibitory activity for the ROS overproduction was observed, and the ROS content wase markedly decreased when the cells were pretreated with 50, 100 and 200 µg/mL of *K. coccinea* extracts compared to the H_2_O_2_ group, and the ROS content decreased as the concentration of *K. coccinea* extracts increased. The ROS content of the HUVEC decreased by 61.53% and 51.92%, respectively, when the pretreatment concentration of ZHP and DHP were set at 200 μg/mL, significantly lower than that in the injured group. Thus, it can be seen that the *K. coccinea* extracts can eliminate or inhibit H_2_O_2_-induced ROS production in cells and protect damaged HUVEC cells.

### 3.5. Effect of K. coccinea Extracts on MDA of HUVEC after H_2_O_2_-Induced Injury 

In the process of degradation of the lipid peroxides, the content of MDA is related to the level of oxidative stress, and excessive free radicals will increase the content of MDA, subsequently leading to the destruction of the structure and function of cell membrane and accelerating the aging of cell membrane. As can be seen from Figure 3a, cells that were exposed to H_2_O_2_ stress had a significant increase in intracellular MDA (nearly 1.74-fold vs. the control group). On the contrary, the overproduction of MDA was significantly inhibited when the cells were pretreated with different sample concentrations, particularly at 200 μg/mL, the MDA content in the DHP and ZHP groups decreased by 91.04 and 53.80%, respectively, which was much lower than that in the H_2_O_2_ group. This was probably due to the flavonoids that have lipophilic properties, so they can remove the lipid peroxides in the phospholipid bilayer of cell membranes. Thus, *K. coccinea* phenolics can play a protective role on the cell membrane to effectively inhibit the generation of MDA and reduce the degree of oxidative damage of HUVEC cells.

### 3.6. Effect of K. coccinea Extracts on SOD of HUVEC after H_2_O_2_-Induced Injury

As the key antioxidant enzyme in the body, SOD is the enzyme that reacts to oxygen free radicals and has the strongest reaction to oxidative stress. As shown in Figure 3c, when the HUVEC was treated with 40 µmol/mL of H_2_O_2_, the SOD activity had approximately 57% of reduction, which was notable lower than that in the control group, suggesting that the HUVEC cells had been significantly injured by oxidation. However, when pretreated with polyphenolics extracts at 50~200 µg/mL, the SOD activity was significantly increased with an obvious dose-dependent relationship. In particular, after 200 μg/mL of epicarp extracts were pretreated, the SOD activity in ZHP and DHP groups was 1.9 and 1.7 times that of the H_2_O_2_ group, respectively, or 70~90% of the control group. These results indicate that *K. coccinea* phenolics extracts have obvious protective effects on cells against H_2_O_2_-induced oxidative stress.

### 3.7. Effect of Extracts on GR of HUVEC after H_2_O_2_-Induced Injury 

GR is a widely available oxidoreductase, which can catalyze NADPH to reduce GSSG to GSH, and plays a key role in the removal of reactive oxygen species in the oxidative stress reaction. Compared with the control group, the GR activity was dramatically decreased to 67.75% under the treatment of 40 µmol/mL of H_2_O_2_ (Figure 3d). Whereas, after pretreatment with ZHP and DHP, the GR activity sharply increased with a dose-dependent matter. Especially at the concentration of 200 μg/mL, the GR activity reached 98.68% and 89.53%, which was 1.5 and 1.3 times that of the H_2_O_2_ group, respectively. These results also indicate that the preventive model is sufficiently effective to relieve oxidative stress, even at lower concentrations and shorter pre-exposure to these compounds. The results also suggest that, even at lower concentrations and shorter pre-pretreatment, *K. coccinea* phenolics extracts can effectively reduce the HUVEC damage caused by oxidative stress.

### 3.8. Phenolics Composition of K. coccinea 

**Figure 4:**
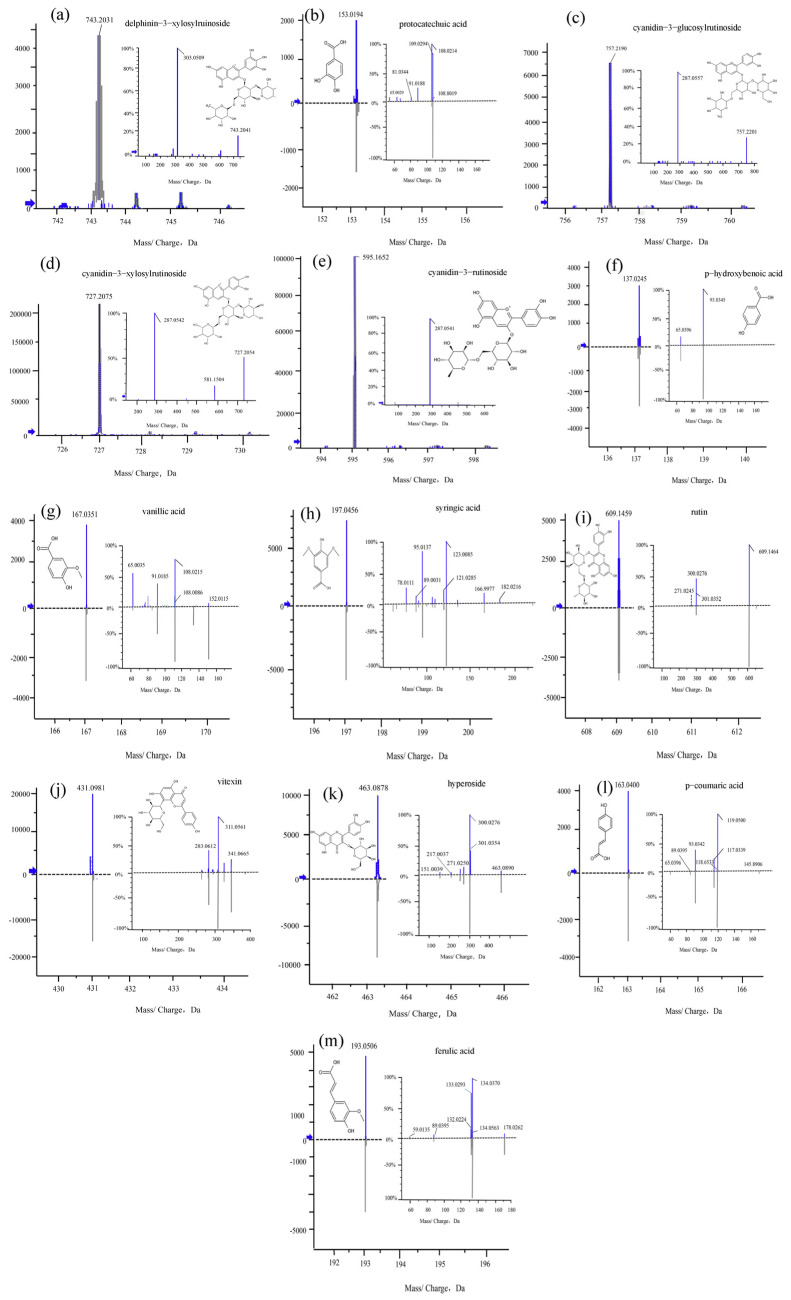
QTOF-MS/MS mass spectra of fragment ion and chemical structures of delphinin-3-xylosylrutinoside (**a**); protocatechuic acid (**b**); cyanidin-3-glucosylrutinoside (**c**); cyanidin-3-xylosylrutinoside (**d**); cyanidin-3-rutinoside (**e**); p-hydroxybenzoic acid (**f**); vanillic acid (**g**); syringic acid (**h**); rutin (**i**); vitexin (**j**); hyperoside (**k**); p-coumaric acid (**l**); ferulic acid (**m**).

The phenolic profiles of the two kinds of *K. coccinea* were performed based on optimized UPLC-QTOP-MS/MS methods. The individual phenolics compounds were identified by comparing the retention times and fragment ions with those of the respective standards (Table 2). Samples were tested in both positive and negative modes, but results were better in the negative mode. Thirteen phenolic compounds were identified in *K. Coccinea* (Figure 4). Among them, seven compounds belong to flavonoids (delphinidin-3-xylosylrutinoside, cyanidin-3-glucosylrutinoside, cyanidin-3-xylosylrutinoside, cyanidin-3-rutinoside, rutin, vitexin, hyperoside), and six compounds (*p*-coumaric acid, ferulic acid, vanillic acid, hydroxybenzoic acid, protocatechuic acid, syringic acid) belong to phenolic acids. 

Compound 1 displayed molecular ion *m*/*z* 743.20 [M+H]^+^ and fragment ion at *m*/*z* 303.05. The 303.05 [M+H]^+^ was generated after the loss of glucose moiety (162 Da), xylose glycosyl (132 Da) and rhamnose moiety (146 Da) from the 743.20 [M+H]^+^, according to the fragment ion peak characteristics and the results reported by Toki [19] and Hao et al. [20]. Compound 1 was tentatively identified as delphinidin-3-xylosylrutinoside.

Compound 2 exhibited [M-H]^−^ ion at *m*/*z* 153.01. Sequential fragmentation gave origin to product ions at *m*/*z* 109.02,108.02 and 91.01. The fragment at *m*/*z* 109.02 [M-H-44]^−^ corresponded with the loss of one molecule of CO_2_ from *m*/*z* 153.01, while *m*/*z* 91.01 [M-H-44-18]^−^ was generated by losing one molecule of H_2_O from *m*/*z* 109.02. When compared to the standard information and literature [21], compound 2 was identified as protocatechuic acid.

Compound 3 displayed a molecular ion *m*/*z* 757.22 [M+H]^+^ and a fragment ion at *m*/*z* 287.05 which corresponded to the cyanidin aglycone. The 287.05 [M+H]^+^ was generated after the loss of two glucose moiety (162 Da) and rhamnose moiety (146 Da) from the *m*/*z* 757.22 [M+H]^+^, which suggested that the compound was one of the anthocyanins in centaurea cyanus. These results were consistent with the characteristic ion of sour cherry [22] and mulberry [23]. Therefore, compound 3 was identified as cyanidin- 3-glucosylrutinoside.

Compound 4 displayed a molecular ion *m*/*z* 727.21 [M+H]^+^ and a fragment ion at *m*/*z* 287.05, which was generated after the loss of glucose moiety (162 Da), xylose moiety (132 Da) and rhamnose moiety (146 Da) from the *m*/*z* 727.21 [M+H]^+^, suggested that the compound was one of anthocyanins in centaurea cyanus. This fragmentation pattern coincides with black raspberry [24] and omija (*Schizandra chinensis*) fruit [25]. Therefore, compound 4 was identified as cyanidin-3-xylosylrutinoside.

Compound 5 displayed a molecular ion *m*/*z* 595.17 [M+H]^+^ and a fragment ion at *m*/*z* 287.05. This fragment was assumed to be the result of the loss of glucose moiety (162 Da) and rhamnose moiety (146 Da) from the *m*/*z* 595.17 [M+H]^+^, suggesting that the compound was one of the anthocyanins in centaurea cyanus, and when compared with the mass spectrum characteristic found in sour cherry [22] and cranberry [26]. Thus, compound 5 was identified as cyanidin-3-rutinoside.

Compound 6 displayed [M-H]^−^ ion at *m*/*z* 137.02 and two fragment ions at *m*/*z* 93.03 and 65.03. The fragment ion at *m*/*z* 93.03 [M-H-44]^−^ was generated by losing one molecule of CO_2_ from *m*/*z* 137.02. Further MS^n^ fragmentation gave origin to product ion at *m*/*z* 65.03 [M-H-44-28]^−^ by the loss of one molecule of CO. When compared with the standard information and literature [27], compound 6 was identified as p-hydroxybenzoic acid.

Compound 7 exhibited molecular ion at *m*/*z* 167.03 [M-H]^−^ with three fragment ions at *m*/*z* 152.01, 108.02 and 91.01. The molecular ion *m*/*z* 167.03 loss of one molecule of CH_3_ generated *m*/*z* 152. 01 [M-H-15]^−^, which further lost one molecule of CO_2_ giving *m*/*z* 108.02 [M-H-15-44]^−^, and then further lost one molecule of C_2_H_3_ giving *m*/*z* 91.01 [M-H-15-44-27]^−^. When compared with the standard information and literature [27], compound 7 was identified as vanillic acid.

Compound 8 generated a molecular ion at *m*/*z* 197.04 [M-H]^−^ and two fragments, one at *m*/*z* 182.02 and the other at *m*/*z* 123. The first fragment was generated as *m*/*z* 197.04, lost one molecule of CH_3_ and formed fragment ion *m*/*z* 182.02 [M-H-15]^−^. The *m*/*z* 123 resulting from the *m*/*z* 182.02 loss of one molecule of CH_3_ and CO_2_. When compared with the standard information and literature [27], compound 8 was identified as syringic acid.

Compound 9 exhibited molecular ion of *m*/*z* 609.15 [M-H]^−^ with three fragment ions at *m*/*z* 301.03, 300.02 and 271.02. Among these fragments, the *m*/*z* 301.03 was generated as aglycone Sour hY_0_]^−^ resulting from parent ion *m*/*z* 609.15 then lost the rutinose moiety, which further lost one molecule of H_2_CO to yield *m*/*z* 271.02. Compound 9 was identified as rutin, based on the standard information and literature [28].

Compound 10 showed a parent ion *m*/*z* 431.1 and two fragment ions at *m*/*z* 341.06 and 311.05. The fragment ion at *m*/*z* 341.06 [M-H-90] and *m*/*z* 311.05 [M-H-120] were produced by the loss of C_3_H_6_O_3_ and C_4_H_8_O_4_ which resulted from the cleavage of sugar chains, respectively. Fragment ions *m*/*z* 283 were generated as *m*/*z* 341continued to lose one molecule of H_2_O and CO. When compared with the standard information and literature [29], compound 10 was identified as vitexin.

Compound 11 exhibited a molecular ion at *m*/*z* 463.09 [M-H]^−^ and two fragment ions at *m*/*z* 301.03 and 300.02. The fragment ion at *m*/*z* 301.03 was generated as aglycone [Y_0_]^−^ resulting from the molecular ion loss at *m*/*z* 162.16, which corresponded with the loss of the galactopyranose moiety. Fragment ions *m*/*z* 300.02 were generated as [Y_0_-H]^−^. When compared with the standard information and literature [30], compound 11 was identified as hyperoside.

Compound 12 showed a [M-H]^−^ at *m*/*z* 163.04 and gave three fragment ions at *m*/*z* 119.05, 93.03 and 65.03. The fragment ion at *m*/*z* 119.05 [M-H-44]^−^ was generated by the loss of one molecule of CO_2_ from *m*/*z* 163.04. The fragment ion at *m*/*z* 93.03 [M-H-56-14]^−^ resulted from the loss of two molecules of CO and one molecule of CH_2_ from *m*/*z* 163.04. When compared with the standard information and literature [27], compound 12 was identified as *p*-coumaric acid.

Compound 13 generated the molecular ion *m*/*z* [M-H]^−^ 193.05 and three fragment ions at *m*/*z* 178.02, 134.05 and 133.02. The fragment ion at *m*/*z* 178.02 [M-H-15]^−^ corresponded to the loss of one molecule of CH_3_ from *m*/*z* 193.05, which further lost one molecule of CO_2_ giving *m*/*z* 134.05 [M-H-15-44]^−^. When compared with the standard information and literature [27], compound 13 was identified as ferulic acid.

### 3.9. Quantitation of Phenolic Compounds Content in Polyphenol Extracts

Concentrations of phenolics were determined from peak area measurements in comparison to a standard curve, except anthocyanin. As there was a lack of corresponding standards, these anthocyanins with complex glycosylation were quantified based on cyanidin-3-glucoside. The mass concentration of each phenolic compounds in *K. coccinea* are summarized in Table 2. Amongst them, anthocyanin were the predominant phenolic compounds in *K. coccinea* epicarp with the value of 1903.88 mg Gy-3-G/100 g in ZHP and 480.34 mg Gy-3-G/100 g in DHP, approximately accounting for 60% and 47% of total phenolics amount in ZHP and DHP, respectively, which was significantly higher than the proportions of hydroxybenzoic acids (14~49%), hydroxycinnamic acids (1~3%) and non-anthocyanins flavonoids (1~25%). Amongst anthocyanins, cyanidin-3-xylosylrutinoside and cyanidin-3-rutinoside have been indicated as the dominant components present in *K. coccinea*, in particular cyanidin-3-xylosylrutinoside was the most abundant anthocyanin. These results are consistent with data obtained by Hao et al. [20]. In this paper, there was 1423.38 ± 29.68 mg Gy-3-G/100 g and 148.37 ± 1.39 mg Gy-3-G/100 g in ZHP and DHP, accounting for 74.76% and 30.89% of the total amount of anthocyanins, respectively. The present results were also significantly higher than that of the Chinese dwarf cherry (0.42 ± 0.11 mg Gy-3-G/kg) [31] and 11.22 mg/100 g in redcurrant [32]. Cyanidin-3-rutinoside might be the secondly highest anthocyanin in *K. coccinea* extracts with the value of 264.59 ± 1.80 and 221.76 ± 6.83 Gy-3-G/100 g in ZHP and DHP, respectively, significantly higher than that that of the *Rubus coreanus* with 3.5~78 mg Gy-3-G/100 g [33], also higher than that of Ribes, Aronia, and Sambucus with the value of 5.83 mg/100 g and cloudberry (wild) with the value of 1.86 mg Gy-3-G/100 g [34]. The cyanidin-3-glucosylrutinoside content in *K. coccinea* extracts was 117.85 ± 2.99 mg Gy-3-G 100/g in ZHP and 94.31 ± 1.42 mg Gy-3-G/100g in DHP, which was higher than that of in *Raspberry-Rubus* idaeus L with 27.95 mg Gy-3-G 100/g [34], and comparable to those in redcurrant reported by the authors in [35] and in sour cherry reported by the authors in [36] (7.64 mg Gy-3-G/100 g and 109.70 mg Gy-3-G/100 g, respectively). Another minor amount of anthocyanin was delphinidin 3-xylosylrutinoside with the value of 98.09 ± 0.67 mg Gy-3-G/100 g in ZHP and 15.9 ± 0.49 mg Gy-3-G/100g in DHP. It was rarely reported in fruits and only found in the petals of linum grandiflorum [19].

The total amount of non-anthocyanin flavonoids include two flavonols (rutin, hyperoside) and one flavone (vitexin)in *K. coccinea* was up to 805.55 mg/100 g. Among them, rutin accounted for 98.70% of the total amount of total flavonoids. To the best of our knowledge, these flavonoids have not been reported so far, in *K. coccinea*. The highest level of phenolic acid observed in *K. coccinea* was protocatechuic acid with the value of 369.71 ± 2.70 mg/100 g in DHP, which represented 35.93% of the total of phenolic compounds, and slightly higher than that of ZHP with the value of 350.74 ± 2.58 mg/100 g. Hydroxybenzoic acid and vanillic acid were present at comparable levels (42~72 mg/100 g) in DHP and ZHP. Traces of *p*-coumaric acid, ferulic acid and syringic acid were all detected in these two kinds of *K. coccinea*.

Interestingly, the content of individual phenolic acids in ZHP were all lower than that of DHP, whereas the content of all anthocyanins, rutin and heperoside in ZHP exhibited higher levels than that of DHP. These concentration differences were supposed to be related to the visual color, as the darker the color, the higher were the levels of anthocyanins. The present difference in phenolics’ quantitation results, combined with those of radical scavenging activities, indicate that these biological activities are most likely ascribed to the dominant phenolic compounds such as cyanidin-3-xylosylrutinoside, cyanidin-3-rutinoside, rutin, protocatechuic acid and hyperoside in *K. coccinea* extracts, especially for cyanidin-3-xylosyl-rutinoside, which was nearly ten times higher than DHP extract. Kim et al. [25] reported that the cyanidin-3-xylosylrutinoside from Omija pigment mostly explained 86% (DPPH) and 98% (ABTS) of total antioxidant activity. Im et al. in [37] reported that cyanidin-3-xylosylrutinoside, yanidin-3-rutinoside, cyanidin-3-sambubioside and cyanidin-3-glucoside were dominant components and contributors to the antioxidant capacity of ripe *R. coreanus Miquel* fruits for the protective effect on neuronal PC-12 cells induced by H_2_O_2_ [37]. The probable protective mechanism for cyanidin glycosides, was proved to possess the effect on oxygen species (ROS)-with the dependent activation of p38 MAPK and JNK [38]. Rutin, as a popular flavonoid, also showed a greater protective effect on the injured HUVECs caused by H_2_O_2_ through a decrease in the level of malondialdehyde (MDA), lactate dehydrogenase (LDH), and an increase in the level of nitrogen oxide (NOS) [39], or a reduction in the ROS, calpain and ceramide levels in mouse kidneys [40]. It was also reported that rutin could upregulate NRF2-mediated endogenous antioxidant responses by oxidizing to quinone to protect HUVECs from oxidative stress induced by H_2_O_2_ [41]. Protocatechuic acid as one of the primary metabolites of cyanidin3-glucoside and cyanidin-3-rutinoside, also showed protective effects on hepatotoxicity induced by cisplatin in mice, through reducing MDA and NO levels and increasing GSH and SOD levels [42], could also reduce palmitic acid-induced oxidative damage to human umbilical vein endothelial cells (HUVECs) or high-fat diet-induced aortic oxidative damage in mice [43,44]. Therefore, a better biological activity of ZHP might be partially attributed to these compounds.

From the discussion above, these phenolic components constitute the material basis against oxidative stress activity for *K. coccinea*, in spite of the detailed molecular mechanisms remaining to be clearly studied. However, the present study could be the first to demonstrate that the anthocyanin-rich phenolics of *K. coccinea* positively affect SOD and GR production as part of the cellular antioxidant mechanism. The results showed that this fruit is an excellent source of phenolic compounds with important antioxidant potential, which provides an important option for the pharmaceutical industry, or as a functional food to develop new natural plant products to combat oxidative stress.

## 4. Conclusions

In the present study, a validated UPLC-QTOF-MS/MS method was employed for the rapid simultaneous quantitative determination of phenolic compounds in *K. coccinea.* Thirteen phenolic compounds were successfully identified and quantified including anthocyanins, flavan-3-ols, flavonols and phenolic acids. For the first time to our knowledge, vitexin, rutin and heperoside were found for the first time in *K. coccinea*. The anthocyanin-rich phenolic extracts of *K. coccinea.* not only have chemical antioxidant activity, but also can reduce the overproduction of ROS and NO and enhance the expression of antioxidant enzymes SOD and GR in cell bioassay. Our data provided evidence on the potential of *K.* *coccinea*. as a rich source of natural antioxidant molecules.

## Figures and Tables

**Table 1:** TPC, TFC, TAC and antioxidant activity of ZHP and DHP.

Sample	TPC (mg GAE/g)	TFC (mg CE/g)	TAC (mg Gy-3-G/g)	DPPH (μmol TE/g)	ABTS (μmol TE/g)	FRAP (μmol TE/g)
ZHP	2.56 ± 0.33 ^b^	5.94 ± 0.30 ^a^	0.71 ± 0.03 ^a^	100.53 ± 2.93 ^a^	68.70 ± 1.24 ^a^	115.23 ± 3.48 ^a^
DHP	3.51 ± 0.14 ^a^	4.33 ± 0.45 ^b^	0.20 ± 0.02 ^b^	111.57 ± 3.10 ^a^	54.96 ± 1.12 ^b^	100.04 ± 2.75 ^b^

Results are expressed as the mean ± SD (*n* = 3), (a), (b) Means with different letter within the same column indicate statistical differences (*p* < 0.05).

**Table 2:** Compositions and content of phenolic compounds from *Kadsura coccinea* epicarp tested by UPLC-QTOF-MS/MS.

Rention Time (min)		Molecular Weight (M.W)	[M-H]^−^/[M-H]^+^ (*m*/*z*)	Characteristic Ion (*m*/*z*)	Identified Compounds	Compounds Content (mg/100 g)	
						ZHP	DHP
1	4.372	742	743 *	303.05	delphinin-3-xylosylrutinoside	98.09 ± 0.67	15.90 ± 0.49
2	4.387	154	153	109.02, 108.02, 91.01	protocatechuic acid	350.74 ± 2.58	369.71 ± 2.70
3	4.444	756	757 *	287.05	cyanidin-3-glucosylrutinoside	117.85 ± 2.99	94.31 ± 1.42
4	4.535	726	727 *	287.05	cyanidin-3-xylosylrutinoside	1423.38 ± 29.68	148.37 ± 1.39
5	4.601	594	595 *	287.05	cyanidin-3-rutinoside	264.59 ± 1.80	221.76 ± 6.83
6	4.896	138	137	93.03, 65.03	*p*-hydroxybenzoic acid	42.00 ± 4.83	72.09 ± 1.70
7	5.286	168	167	152.01, 108.02, 91.01	vanillic acid	49.44 ± 3.72	51.31 ± 2.82
8	5.450	198	197	182.02, 123	syringic acid	11.15 ± 0.98	12.85 ± 1.37
9	5.601	610	609	301, 300, 271	rutin	795.44 ± 15.26	7.27 ± 0.49
10	5.952	432	431	341.06, 311.05, 283.06	vitexin	1.75 ± 0.11	1.81 ± 0.50
11	6.027	464	463	301.03, 300.02	hyperoside	8.36 ± 0.56	1.61 ± 0.24
12	6.095	164	163	119.05, 93.03, 65.03	*p*-coumaric acid	19.15 ± 1.97	23.64 ± 2.23
13	6.667	194	193	178.02, 134.05, 133.02	ferulic acid	6.14 ± 0.95	8.21 ± 0.13

Results are expressed as the mean ± SD (*n* = 3). The band * indicates detection in positive ion mode.

## Data Availability

Not applicable.
